# Hybrid Strategies for CTO PCI: A Systematic Review and Meta-Analysis of Antegrade and Retrograde Techniques

**DOI:** 10.3390/life15111739

**Published:** 2025-11-12

**Authors:** Andrei-Mihnea Rosu, Maria-Daniela Tanasescu, Theodor-Georgian Badea, Emanuel-Stefan Radu, Eduard-George Cismas, Alexandru Minca, Oana-Andreea Popa, Luminita-Florentina Tomescu

**Affiliations:** 1 Department of Cardiology, Prof. Dr. Agrippa Ionescu Emergency Hospital, 077015 Balotesti, Romania; andrei-mihnea.rosu@drd.umfcd.ro (A.-M.R.); radu.emanuel@dcti.ro (E.-S.R.); eduard-george.cismas@rez.umfcd.ro (E.-G.C.); popa.oana@dcti.ro (O.-A.P.); 2 Department of Semiology, Emergency University Hospital, Carol Davila University of Medicine and Pharmacy, 022328 Bucharest, Romania; alexandru.minca@umfcd.ro; 3 Department of Radiology, Prof. Dr. Agrippa Ionescu Emergency Hospital, 077015 Balotesti, Romania; luminita.tomescu@umfcd.ro; 4 Department of Radiology, Carol Davila University of Medicine and Pharmacy, 022328 Bucharest, Romania

**Keywords:** chronic total occlusion, percutaneous coronary intervention, antegrade dissection and re-entry, retrograde approach, intravascular imaging, hybrid algorithm

## Abstract

**Background**: Chronic total occlusion percutaneous coronary intervention (CTO PCI) is a complex revascularization procedure requiring advanced techniques to ensure procedural success and safety. Hybrid strategies combining antegrade dissection/re-entry (ADR) and retrograde approaches have become increasingly adopted in contemporary practice. **Objectives**: To systematically review and synthesize evidence comparing outcomes of ADR and retrograde CTO PCI techniques, with pooled estimates of success rates and adverse events. **Methods**: This review followed PRISMA 2020 guidelines. We searched PubMed, Cochrane CENTRAL, and Google Scholar for studies published between January 2015 and June 2025. Eligible studies included randomized controlled trials and observational studies reporting outcomes of ADR and/or retrograde CTO PCI. Data extraction was performed by two independent reviewers. Risk of bias was assessed using the Newcastle–Ottawa Scale and the Cochrane RoB 2.0 tool. A random-effects meta-analysis was conducted for consistently reported outcomes. **Results**: Twenty studies encompassing over 87,000 CTO PCI procedures were included. Pooled analysis of 16 studies demonstrated a technical success rate of 83.4% and a procedural success rate of 84.6%. The in-hospital major adverse cardiac event (MACE) rate was 3.3%. Hybrid strategies integrating ADR and retrograde approaches yielded the highest success rates (86–91%) with acceptable safety profiles. Use of adjunctive tools such as IVUS, dual arterial access, and re-entry devices was associated with improved outcomes. **Discussion**: Hybrid CTO PCI techniques are safe, effective, and reproducible across diverse clinical settings. When performed by experienced operators using modern adjuncts, these strategies provide durable benefits and should be considered standard for complex occlusions. Limitations include variation in study quality, heterogeneous procedural definitions, and lack of long-term data in several cohorts.

## 1. Introduction

Chronic total occlusions (CTOs) are defined as coronary artery lesions with Thrombolysis in Myocardial Infarction (TIMI) grade 0 flow for a duration of at least three months, typically affecting vessels larger than 2.5 mm in diameter [1]. CTO PCI refers to percutaneous coronary intervention specifically aimed at recanalizing these long-standing occlusions using one or a combination of antegrade wire escalation (AWE), antegrade dissection and re-entry (ADR), retrograde wire escalation (RWE), or retrograde dissection and re-entry (RDR) techniques, often supported by intravascular imaging [2]. These approaches are distinct from conventional PCI and are considered among the most technically complex procedures in interventional cardiology. The hybrid strategy refers to a dynamic decision-making framework in which operators systematically select and shift between antegrade and retrograde techniques (AWE, ADR, RWE, RDR) based on real-time angiographic features such as proximal cap ambiguity, lesion length, collateral channel quality, and prior failed attempts.

CTOs are encountered in 15–35% of patients undergoing coronary angiography for known or suspected coronary artery disease (CAD) [1,3]. In specific populations, such as those with prior coronary artery bypass grafting (CABG), CTOs are present in over 50% of native coronary vessels, whereas in ST-elevation myocardial infarction (STEMI), prevalence is estimated at 9–11% [4,5]. However, the true prevalence remains underreported due to asymptomatic or conservatively managed cases.

CTO lesions share risk factors with general coronary atherosclerosis, including diabetes, hypertension, dyslipidemia, tobacco use, chronic kidney disease, postmenopausal status, and family history of premature CAD [6]. Despite a high burden of angina symptoms, viable myocardium, and preserved LV function, fewer than 10% of patients with CTO undergo PCI, as shown in large registry data [3].

Given the procedural difficulty, the J-CTO score was developed to help operators estimate the likelihood of successfully crossing a CTO within 30 min. It incorporates five variables: blunt stump, occlusion length ≥20 mm, calcification, bending >45°, and prior failed attempt. Crossing success decreases as the score increases, from 88% for score 0 to 10% for ≥3 [7].

Technical refinements over the last decade—including hybrid algorithms, improved operator training, intravascular imaging, and specialized microcatheters—have improved success rates to over 85% in experienced centers [3,8]. The 2021 ACC/AHA/SCAI Guidelines recommend CTO PCI as a Class IIb indication in appropriately selected symptomatic patients treated by experienced operators [8].

Given the persistent variability in practice patterns and procedural outcomes across CTO PCI strategies, a systematic comparison is warranted. To our knowledge, no prior review has comprehensively synthesized the success rates, complication profiles, and technical evolution of antegrade dissection/re-entry and retrograde techniques within the hybrid framework. This review aims to systematically evaluate and compare procedural characteristics, outcomes, and safety of ADR and retrograde CTO PCI approaches, using data from 2015–2025.

## 2. Materials and Methods

This review was conducted in accordance with the PRISMA 2020 guidelines and the Cochrane Handbook for Systematic Reviews of Interventions (version 5.1.0). A completed checklist and flow diagram are provided in the Supplementary Materials.

### 2.1. Eligibility Criteria

We included randomized and observational studies reporting outcomes of CTO PCI using either antegrade dissection and re-entry (ADR) techniques (e.g., ADR, AFR, RFR, HDR, BASE, Side-BASE) or retrograde approaches (e.g., CART, reverse CART, retrograde wire escalation, confluent ballooning). Studies had to report at least one procedural outcome (e.g., technical success, complication rate). We excluded animal studies, in vitro models, case reports, editorials, conference abstracts, and non-English publications.

### 2.2. Search Strategy

A systematic search was performed using PubMed, Cochrane CENTRAL, and Google Scholar from January 2015 to June 2025. All sources were last searched on 30 June 2025. The search combined keywords such as “chronic total occlusion,” “CTO,” “PCI,” “antegrade dissection,” “retrograde PCI,” “wire escalation,” “ADR,” “AFR,” “RFR,” “CART,” and related procedural terms. Boolean operators (AND, OR) were used for optimized retrieval. Additional references were identified through manual screening of bibliographies from included studies and relevant reviews. Detailed search strings for PubMed, Cochrane CENTRAL, and Google Scholar are included in Supplementary Table S1.

### 2.3. Study Selection

**Figure 1:**
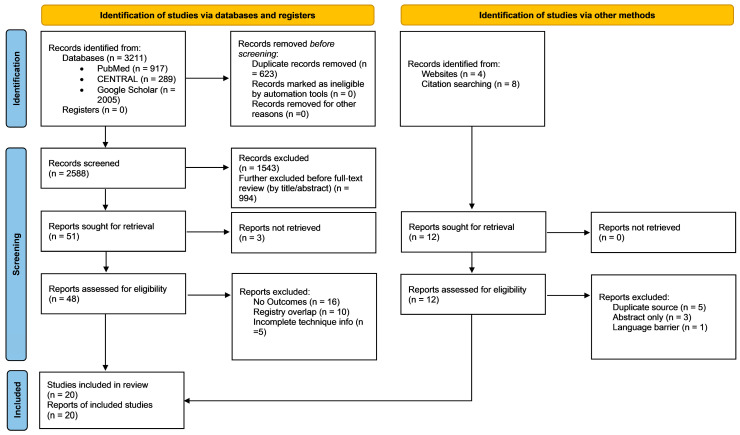
PRISMA 2020 flow diagram detailing the study selection process.

Two reviewers independently screened all titles and abstracts, followed by full-text assessment of eligible articles. Discrepancies were resolved through consensus with a third reviewer. A PRISMA flow diagram outlining the selection process is presented in Figure 1.

### 2.4. Data Extraction and Management

Data extraction was performed using a standardized spreadsheet by two independent reviewers. Extracted data included:

- Study characteristics: year, country, design, sample size;
- Patient population: age, sex, comorbidities (when reported);
- Procedural strategy: ADR, RWE, RDR, hybrid;
- Devices used: IVUS, CrossBoss, Stingray, microcatheters;
- Primary outcomes:
  – Technical success (successful CTO crossing with restoration of TIMI flow and <30% residual stenosis);
  – Procedural success (technical success without in-hospital MACE);
  – In-hospital MACE: death, MI, stroke, or target vessel revascularization.
- Secondary outcomes: revascularization, restenosis, perforation, tamponade, mortality.

Funding sources, conflict of interest data, and core-lab adjudication were not consistently reported and, therefore, not extracted.

Missing or unclear values were recorded as “Not Reported.” No data imputation was performed.

A third reviewer independently validated all extracted data for accuracy and completeness.

### 2.5. Quality Assessment

Risk of bias was assessed using the Newcastle–Ottawa Scale (NOS) for observational studies and the Cochrane RoB 2.0 tool for randomized trials. NOS scores ≥ 7 were considered high quality. RoB 2.0 domains included randomization, deviations from intended interventions, missing data, outcome measurement, and reporting bias. All disagreements were resolved by consensus.

### 2.6. Data Synthesis and Quantitative Analysis

Where definitions were consistent, we performed random-effects meta-analyses using the DerSimonian–Laird method to calculate pooled event rates. The following effect measures were used: pooled proportions (technical success, procedural success, MACE), reported with 95% confidence intervals (CIs).

Between-study heterogeneity was evaluated using I^2^ and τ^2^ statistics. Statistical analyses were performed using Python v3.11, including Statsmodels v0.14, and forest plots were visualized using Matplotlib v3.8.

Studies lacking adequate reporting of key outcomes were excluded from pooled estimates. No imputation was performed.

Sensitivity and subgroup analyses were not conducted due to heterogeneity in study design and limited RCT data.

Risk of reporting bias (e.g., small study effects) was not assessed due to the observational nature of most included studies and the absence of funnel plot symmetry analysis.

Certainty of evidence was not formally assessed using GRADE, given the predominance of non-randomized data and methodological variability.

## 3. Results

### 3.1. Search Results

A total of 3211 records were identified through database searches, including 917 from PubMed, 289 from Cochrane CENTRAL, and 2005 from Google Scholar. An additional 12 records were identified through other sources, including 4 from websites and 8 through citation searching. After removing 623 duplicate records, 2588 records remained for screening. Of these, 1543 were excluded for not meeting eligibility criteria, and a further 994 were excluded prior to full-text assessment. In total, 51 full-text articles were assessed for eligibility, of which 31 were excluded for the following reasons: no relevant outcomes (*n* = 16), registry or cohort duplication (*n* = 10), and incomplete technical detail (*n* = 5). Ultimately, 20 studies were included in the final systematic review and synthesis. The study selection process is illustrated in Figure 1 (PRISMA 2020 flow diagram).

### 3.2. Study Characteristics

This review included 20 studies published between 2015 and 2024, encompassing over 87,000 CTO PCI procedures performed across North America, Europe, Asia, the Middle East, and Latin America. The studies represented a spectrum of clinical settings, including randomized controlled trials [9,10], prospective registries [11,12,13], and national databases [14,15,16]. Most were multicenter, observational investigations reflecting real-world CTO PCI practice across both high-volume specialist centers and broad population registries.

The study populations varied widely in size and complexity. The largest datasets originated from national or multinational registries, including the BCIS registry [14] with 28,050 procedures, the ERCTO registry [17,18] with over 17,000 cases, and the VA CART program [15] with 2516 CTO lesions treated within the U.S. Veterans Affairs system.

Technique-focused registries such as PROGRESS-CTO [19,20,21,22] and OPEN-CTO [13] provided detailed procedural analyses, while single-center data were reported from Asan Medical Center in Korea [23] and the RAIAN registry in Iran [24].

Across all studies, the mean patient age ranged from the mid-50s to early 70s, with a predominance of male patients and a high prevalence of hypertension, diabetes, and multivessel disease. Later studies included more complex lesions, such as long occlusions, severe calcification, and ambiguous proximal caps.

Most studies evaluated strategies structured around the Hybrid Algorithm, integrating four core crossing techniques: Antegrade Wire Escalation (AWE), Antegrade Dissection and Re-entry (ADR), Retrograde Wire Escalation (RWE), and Retrograde Dissection and Re-entry (RDR). Early hybrid studies like RECHARGE [11] and PROGRESS-CTO [19,20] demonstrated dynamic intra-procedural switching among strategies to optimize outcomes. Later analyses [21,22] highlighted the evolving role of ADR, noting its gradual decline in favor of wire-based re-entry techniques. European registry data [17,18] confirmed an increasing reliance on retrograde strategies—from 10% in 2008 to nearly 30% by 2015—while ADR use remained limited. In contrast, Asian cohorts [12,23] reported high procedural success using both antegrade and retrograde approaches, particularly in expert centers, with retrograde success rates of 78–87%.

Device utilization varied by region. CrossBoss and Stingray systems were commonly employed in ADR and hybrid strategies within North American and European registries [11,19,25], whereas IVUS-guided wiring and microcatheter techniques were favored in Japan and Korea [12,23]. The BCIS registry [14] demonstrated that adoption of enabling strategies—dual access, atherectomy, and IVUS—significantly improved technical success rates from 55% to 83% over an eight-year period.

Primary endpoints included technical success, procedural success, and major adverse cardiac events (MACEs)—typically encompassing death, myocardial infarction, stroke, or target vessel revascularization. Reported technical success ranged from 70.3% in a developing center [24] to over 90% in specialized or trial settings [10,12,19]. Most studies reported MACE rates between 1.7% and 4.3%, although ADR and retrograde approaches were associated with higher perforation rates [21,26].

Long-term outcomes were available in select studies. Hannan et al. [16] demonstrated a significant mortality benefit for successful CTO PCI at 2.5 years. Kwon et al. [23] reported higher target vessel failure following retrograde procedures over four years. The DECISION-CTO trial [10] confirmed durable angina relief and quality-of-life improvement but no reduction in MACE compared to optimal medical therapy.

Collectively, these data reflect the global evolution of CTO PCI—from simple antegrade wiring to the widespread use of hybrid algorithms combining dissection/re-entry and retrograde strategies. Success rates have increased with operator experience and broader adoption of adjunctive technologies. While most studies emphasized procedural metrics, emerging registry data support the long-term safety and clinical benefit of contemporary CTO PCI—Table 1.

### 3.3. Technical Approaches

Over the past decade, CTO PCI has evolved from a niche, high-risk procedure into a standardized and reproducible intervention supported by structured hybrid algorithms. This evolution integrates AWE, ADR, and retrograde strategies—including reverse controlled antegrade and retrograde tracking (rCART), wire escalation, and confluent balloon techniques—enabling tailored approaches based on lesion complexity and procedural progress. These contemporary strategies, their comparative deployment, and randomized validation are summarized in Table 2 and Table 3.

Randomized data have provided safety validation for CTO PCI techniques. The DECISION-CTO trial [10] demonstrated high procedural success (≈90%) using operator-determined strategies, with quality-of-life improvement post-intervention. Meanwhile, the CrossBoss First Trial [25] compared dedicated dissection/re-entry (ADR with CrossBoss/Stingray) to standard wire escalation, finding comparable technical success (~88%) and confirming that ADR offers procedural parity without increased risk when appropriately applied.

Observational registries have substantially expanded the understanding of real-world strategy usage and operator behavior. The RECHARGE registry [11], one of the earliest European multicenter evaluations of hybrid CTO PCI, formalized the integration of ADR and retrograde strategies into procedural planning. Similarly, the PROGRESS-CTO registry [19,20,21] demonstrated that strategic flexibility—specifically the ability to switch from wire escalation to ADR or retrograde mid-procedure—was decisive in achieving consistent success (>85%), particularly in complex lesions.

Advanced registries have also revealed longitudinal shifts in preferred techniques. A multicenter trend analysis by Rempakos et al. [22] involving 46 centers showed a sharp decline in ADR usage, from 37.9% in 2012 to 14.5% by 2022. This coincided with decreased use of device-based systems like CrossBoss and Stingray, and increased adoption of wire-based re-entry techniques such as STAR and side-BASE. Particularly, ADR was more frequently deployed as a secondary (42.1%) or tertiary (40.2%) strategy, stressing its role as a bail-out maneuver rather than a first-line approach.

Comparative registry data also inform the technical implications of strategy choice. In a PROGRESS-CTO subanalysis [27], ADR showed modestly higher technical success (78%) versus parallel wiring (75%), though associated with significantly higher in-hospital complications. Additionally, ADR was independently associated with more challenging anatomy, including longer lesions, heavy calcification, and ambiguous caps—requiring tools such as CrossBoss, Stingray, and knuckle wire advancement to facilitate re-entry.

Retrograde techniques have become increasingly prominent in high-volume centers, particularly in Asia. The Japanese CTO-PCI Expert Registry [12] and the Asan Medical Center experience [23] emphasized retrograde success rates of 78–87% with systematic IVUS guidance. These centers reported high procedural control with microcatheter-supported channel tracking and meticulous wire manipulation, illustrating the feasibility of complex retrograde interventions when performed by experienced operators. Meanwhile, broader datasets such as the BCIS Registry [14] and ERCTO [17,18] captured the progressive integration of enabling strategies—dual access, atherectomy, and intravascular imaging—alongside hybrid techniques. In BCIS, technical success improved from 56.8% to 83.8% as these adjuncts were adopted. ERCTO data further confirmed increasing retrograde use over time, particularly in Europe, where its application rose from 10% to nearly 30% between 2008 and 2015. The RAIAN Registry [24] from Iran provides further insight into the scalability of hybrid algorithms. Despite resource limitations and lower operator experience, a simplified hybrid strategy achieved 70.3% success, illustrating the translatability of structured techniques to developing programs.

Collectively, data across global studies support a strategic framework in which ADR and retrograde methods are selectively applied based on anatomy, prior failures, and operator expertise. While ADR offers value in re-entry after escalation failure, its use is diminishing due to higher complexity, longer fluoroscopy, and increased perforation risk [22,27]. In contrast, retrograde approaches—especially when guided by IVUS—are increasingly favored in specialized centers.

### 3.4. Procedural Outcomes

Procedural performance across contemporary CTO PCI studies demonstrates high technical success and acceptable safety in both randomized trials and real-world practice. Among the 20 included studies, technical success ranged from 70.3% to 93.1%, while procedural success—defined as angiographic success without in-hospital major adverse cardiac events (MACEs)—averaged 84.6%. Reported in-hospital MACE rates were generally below 4%, and perforation rates ranged from 1% to 3%, increasing to >5% with dissection/re-entry techniques (Table 4).

When stratified by crossing strategy (Table 5), AWE demonstrated the highest and most consistent success (90–93%) with low complication rates (MACE 1.5–2.5%). In contrast, ADR showed lower technical success (75–86%) and higher complication rates (MACE 3–5%; perforation 3–9%), reflecting its use in longer, calcified lesions. RWE achieved 85–88% success with MACE < 4%, while RDR had the lowest success rates (78–83%) and the highest perforation rates (5–7%). Application of hybrid strategies, combining multiple approaches, yielded technical success rates of 86–91% with reliable safety profiles across regions.

The use of adjunctive or “enabling” technologies has further enhanced outcomes. In the BCIS registry [4], cumulative adoption of dual arterial access, intravascular ultrasound (IVUS), and atherectomy increased technical success from 56.8% to 83.8%. Similar improvements were reported in the ERCTO and PROGRESS-CTO registries [17,20], where increased uptake of imaging-guided and hybrid techniques led to steady gains in both success and safety. In particular, national programs in resource-limited settings also reported technical success near 70%, confirming the feasibility of structured hybrid algorithms across healthcare systems [24].

All in all, these data confirm that modern CTO PCI has reached a stage of procedural maturity, with technical success approaching 90% in expert centers, modest complication rates, and consistent reproducibility across techniques, regions, and operator experience levels.

**Figure 2:**
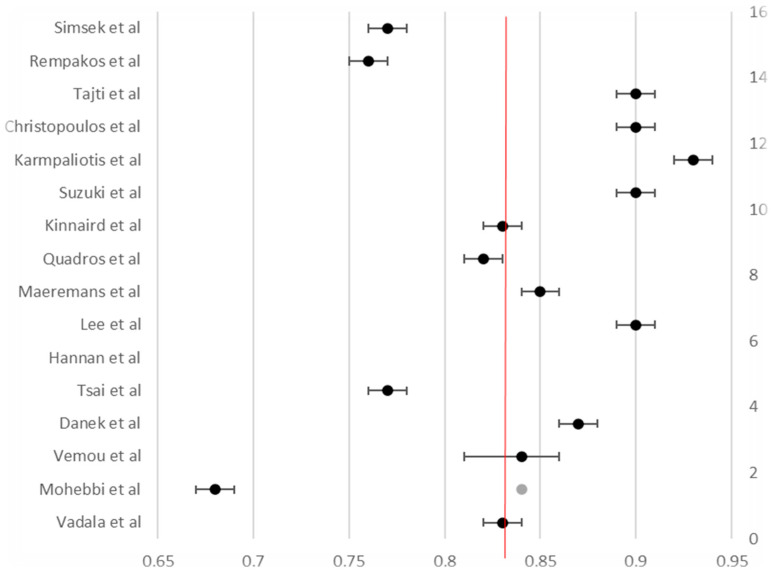
Forest plot of procedural success rates in CTO PCI across 16 studies. The pooled procedural success rate was 83.4%, represented by the red vertical line. Each black dot shows the point estimate from an individual study, with horizontal lines indicating the 95% confidence intervals. The grey dot and its confidence interval reflect the meta-analytic pooled estimate. Minor heterogeneity is noted across study-level success rates.

**Figure 3:**
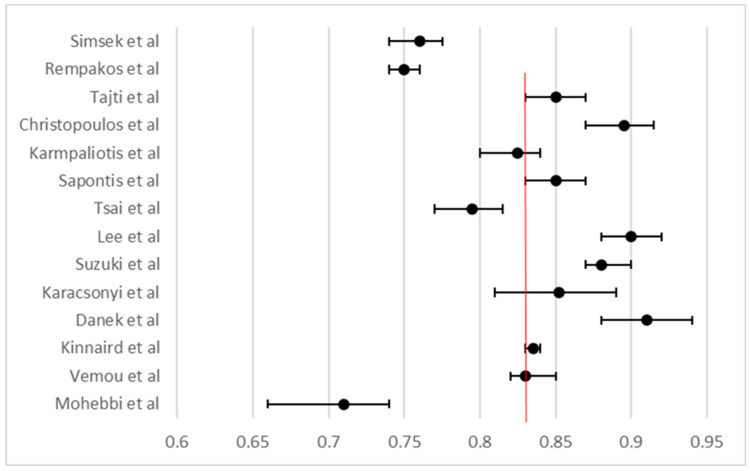
Forest plot of procedural success rates in CTO PCI across 15 studies. The pooled procedural success rate was 83.3%, indicated by the red dashed vertical line. Each black dot represents a study-specific point estimate, and the horizontal bars denote 95% confidence intervals derived from standard errors. Overall, procedural success rates were relatively consistent across studies, with moderate inter-study variability.

**Figure 4:**
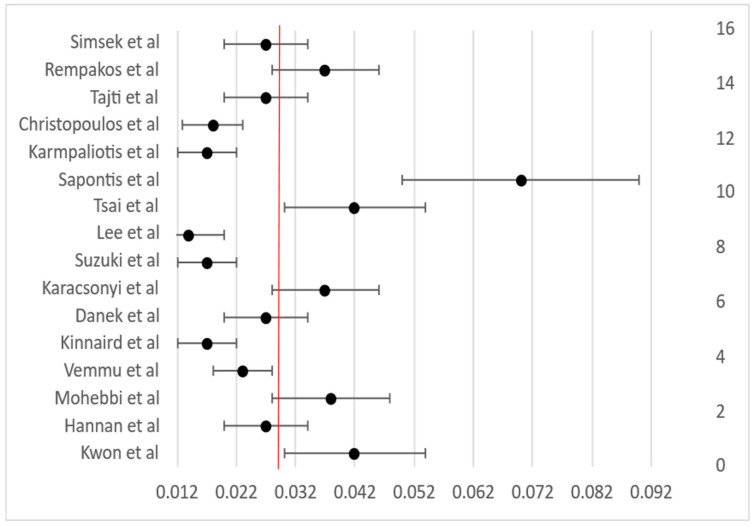
Forest plot of in-hospital MACE rates in CTO PCI across 16 studies. The pooled MACE rate was 2.9%, as indicated by the red dashed vertical line. Horizontal bars represent 95% confidence intervals calculated from study-specific standard errors. Minimal variation was observed between registry-based and single-center studies.

A random-effects meta-analysis of 16 studies, encompassing over 80,000 procedures, yielded a pooled technical success rate of 83.4% (95% CI not shown; see Figure 2), confirming the strength of outcomes across heterogeneous settings. Likewise, meta-analysis of 15 studies showed a pooled procedural success of 84.6% (Figure 3). Across 16 studies, the pooled in-hospital MACE rate was 3.3% (Figure 4), reflecting low periprocedural risk despite the technical complexity of these interventions.

### 3.5. Long-Term Clinical Outcomes

Long-term outcomes were reported in only six of the twenty included studies [10,13,15,16,23,28]. Follow-up ranged from one month to four years. The remaining studies reported only procedural or in-hospital data.

In the DECISION-CTO randomized trial [10] (median follow-up four years), the composite endpoint of death, myocardial infarction, stroke, or repeat revascularization occurred in 22.3% of patients undergoing CTO PCI and 22.4% of those managed with optimal medical therapy. Despite neutral survival findings, PCI produced durable symptom and quality-of-life benefits, with significant improvements in angina frequency and physical limitation scores maintained through 12–36 months.

The VA CART registry [15] (two-year follow-up, *n* = 2394) demonstrated a clear prognostic advantage for successful CTO PCI: mortality was significantly lower (HR 0.67; *p* = 0.023), and subsequent CABG was markedly reduced (HR 0.14; *p* < 0.001). Rates of rehospitalization for MI did not differ between successful and failed cases. The New York State registry [16] confirmed these results, showing lower two-and-a-half-year mortality after successful PCI (6.1% vs. 11.7%; *p* < 0.0001). Failed recanalization independently predicted higher mortality (adjusted HR 1.56, 95% CI 1.19–2.05).

At the Asan Medical Center [23] (four-year follow-up), target vessel failure (TVF) was more frequent after retrograde PCI (17.1%) than antegrade-only PCI (9.4%; *p* = 0.01), largely due to higher reocclusion and repeat revascularization rates. Cardiac death and target-vessel MI were similar between strategies. Predictors of TVF included renal dysfunction (HR 3.33), acute coronary syndrome (HR 1.99), higher J-CTO score (HR 1.23), and smaller stent diameter (HR 0.39).

In-stent CTOs showed higher recurrence risk in the pooled analysis [28] (median 12 months). Compared with de novo CTO PCI, in-stent occlusions had higher MACE (HR 1.31; 95% CI 1.01–1.70) and TVR (HR 1.34; 95% CI 1.00–1.81), though mortality and MI were similar.

Functional outcomes were favorable across all studies with symptom assessments. In the OPEN-CTO registry [13] (one-month follow-up, 94.7% completion), successful PCI produced major improvements in all Seattle Angina Questionnaire domains, including a +26.6-point gain in quality-of-life score, with reductions in angina frequency, dyspnoea, and depression. Improvements were significantly greater after successful versus unsuccessful procedures. These findings mirror the symptomatic gains reported in DECISION-CTO and emphasize the consistent relief of angina and functional limitation following successful recanalization.

### 3.6. Quality Assessment Results

The methodological quality of the 20 included studies was assessed using the Newcastle–Ottawa Scale (NOS) for observational designs and the Cochrane Risk of Bias 2.0 (RoB 2.0) tool for randomized controlled trials. Of the 20 studies, 18 were observational and 2 were randomized trials.

Among the observational studies, 12 were rated as high quality (NOS ≥ 7) based on criteria such as multicenter prospective designs, use of standardized definitions for technical and procedural success, and statistical adjustment for confounding factors. These included: [11,12,13,14,15,16,17,18,19,20,21,24].

The remaining six observational studies [22,23,26,27,28,29] were rated as moderate quality due to at least one of the following limitations: retrospective design, lack of core laboratory adjudication, or incomplete reporting of lesion complexity and long-term follow-up.

The two randomized trials [10,25]—CrossBoss and First Trial—were assessed using the RoB 2.0 tool. Both were judged to be at low risk of bias in the domains of randomization and outcome measurement. However, they exhibited “some concerns” in the domains of deviations from intended interventions and missing outcome data due to crossover between strategies and incomplete long-term reporting.

**Figure 5:**
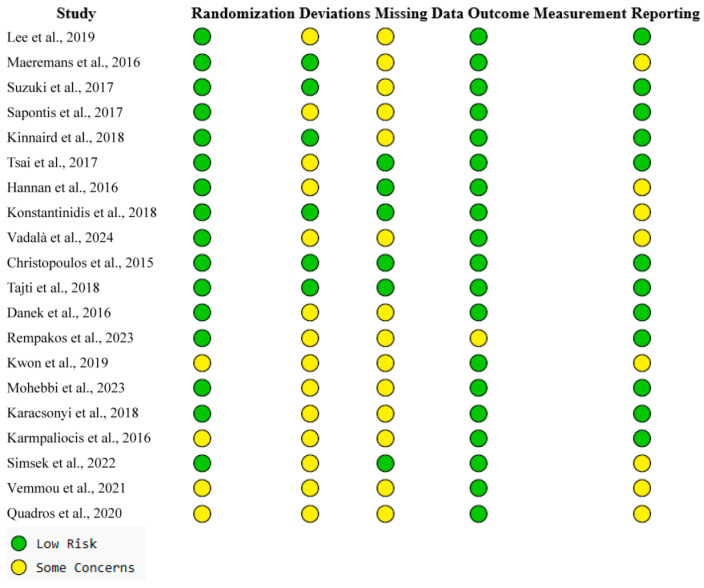
Domain-level Risk of Bias (RoB) assessments for all included studies [10,11,12,13,14,15,16,17,18,19,20,21,22,23,24,25,26,27,28,29].

Overall, the included studies represent a methodologically sound evidence base with a predominance of high-quality prospective observational data. Inter-rater agreement for all quality assessments was high (κ = 0.86), confirming consistency in study grading. A visual summary of domain-level bias assessments is presented in Figure 5.

## 4. Discussion

Early prospective data [11,19,20] established the hybrid algorithm as the procedural cornerstone of modern CTO PCI. These registries demonstrated that intra-procedural flexibility—particularly the ability to switch between antegrade wire escalation, dissection/re-entry, and retrograde approaches—markedly improved procedural efficiency and reproducibility. Strategy switching occurred in up to 42% of procedures [19], emphasizing the algorithm’s dynamic, anatomy-driven nature. Both European and North American datasets confirmed that hybrid adoption elevated technical success to >85% while maintaining acceptable complication rates (2–4%). These findings are in alignment with earlier foundational descriptions of the hybrid approach. Brilakis et al. [30] outlined the algorithm’s core principles, including the dynamic selection of CTO crossing strategies based on four angiographic characteristics—proximal cap morphology, occlusion length, distal target vessel, and collateral suitability. The algorithm encourages early strategy switching, which has been shown to enhance procedural efficiency and safety. Their multicenter evaluations demonstrated that the hybrid approach yields consistently high technical success (90–95%) and low complication rates (1.8–2.0%), especially when performed in high-volume centers with expertise in all three main strategies: antegrade wire escalation, antegrade dissection/re-entry, and retrograde crossing. Schumacher et al. [31] further reinforced the foundational role of the hybrid algorithm in CTO PCI by describing it as a “universal” approach adaptable across lesion types and operator styles. They emphasized that the strategy enables procedural planning to be simplified through the use of four core crossing techniques—AWE, ADR, RWE, and RDR—selected based on three key angiographic parameters: proximal cap ambiguity, distal vessel quality, and interventional collaterals. In addition, they proposed a structured, reproducible framework for CTO interventions that aligns closely with contemporary international registry data and promotes a standardized approach even in centers with varying levels of experience.

A notable trend across contemporary registries is the gradual decline in device-based ADR techniques, particularly those involving CrossBoss and Stingray systems. This shift reflects an increasing preference for wire-based re-entry strategies, such as STAR and side-BASE, which offer comparable success with potentially lower risk profiles and greater procedural flexibility [22,23]. Regional variation also persists: Japanese centers frequently adopt retrograde-first approaches supported by intravascular imaging [12], while European operators tend to limit ADR use and favor hybrid escalation with selective retrograde strategies [18]. 

Temporal data [14,17,18] reinforce this procedural maturation. Between 2008 and 2015, European success rates rose from 79.7% to 89.3%, despite rising lesion complexity and greater use of retrograde strategies. Likewise, increasing implementation of enabling techniques such as dual arterial access, IVUS, and atherectomy improved success from 56.8% to 83.8% in the BCIS dataset [14].

Contemporary studies provide nuanced comparisons between crossing techniques. The PROGRESS-CTO ADR subanalyses [21,22,27] and ERCTO reclassification [18] confirmed that AWE remains the most efficient and safest initial approach, with success rates > 90% and low complication risk. ADR, although essential as a secondary strategy, demonstrated lower success (75–86%) and higher perforation rates (up to 9%), largely due to its use in heavily calcified or long occlusions. These data indicate that ADR should be applied selectively, ideally as part of hybrid escalation rather than a default approach.

The CrossBoss First Trial [25] established the safety and procedural parity of device-based ADR compared with conventional wiring, while Rempakos et al. [22] and Simsek et al. [27] illustrated its declining use in favor of wire-based re-entry techniques such as STAR and Side-BASE. This shift reflects growing operator proficiency and a trend toward less device-dependent re-entry methods. In Europe, ADR adoption remains modest, accounting for <15% of procedures by 2022 [22], while in the U.S. and Asia, its role is increasingly confined to complex bailout situations.

Retrograde strategies have expanded the feasibility of CTO PCI in anatomically challenging lesions. The Japanese Expert Registry [12] and Asan Medical Center series [23] reported retrograde success rates of 78–87% with IVUS-guided precision and low mortality (<0.3%). Although retrograde dissection/re-entry exhibited lower success and higher perforation risk in ERCTO analyses [18], it remains indispensable when antegrade crossing is not feasible.

International datasets underline both the globalization and contextual variability of CTO PCI. The Latin American CTO Registry [29] achieved technical success rates of 82.5%—comparable to Western data—despite the lower availability of specialized devices. Also, the RAIAN Registry [24] from Iran demonstrated successful implementation of the hybrid algorithm with 70.3% success and low in-hospital MACE (3.5%), affirming its transferability to developing programs. However, not all regions have adopted the hybrid strategy uniformly. A nationwide analysis from Poland by Dąbrowski et al. [32] reviewed nearly 850,000 PCI procedures over a ten-year period (2012–2021) and found that CTO PCI accounted for only 2.8% of cases, with no clear increase in adoption despite established international guidelines. Furthermore, use of retrograde techniques declined substantially (from 11.4% to 4.8%), and there was no evidence of widespread implementation of the hybrid algorithm. Their findings emphasize a persistent gap between consensus recommendations and real-world practice, highlighting the need for structured CTO programs, operator certification, and institutional support to facilitate broader integration of algorithm-driven CTO PCI.

Operator experience remains a major determinant of outcomes. The New York State registry [16] demonstrated progressively higher technical success with increasing operator volume (71.2% for high-volume vs. 55.2% for low-volume centers), consistent with findings from earlier global analyses.

Beyond technical metrics, clinical outcomes demonstrate the durable benefits of successful recanalization. The VA CART [15] and New York State [16] registries both reported lower long-term mortality and fewer CABG referrals following successful CTO PCI. In DECISION-CTO [10], despite no difference in major adverse cardiac events between PCI and medical therapy, patients undergoing PCI experienced sustained improvement in angina and physical limitation scores through 36 months. Similarly, the OPEN-CTO registry [13] documented substantial one-month quality-of-life gains (+26.6 points on the Seattle Angina Questionnaire) and reduction in angina frequency, dyspnea, and depressive symptoms, establishing symptom relief and functional recovery as the most reproducible long-term advantages of CTO PCI. Recent evidence continues to clarify the prognostic impact of CTO PCI. A 2024 meta-analysis by Macherey-Meyer et al. [33] pooled data from over 24,000 patients and demonstrated that successful PCI was associated with a statistically significant reduction in all-cause mortality (RR: 0.79, *p* = 0.002) and marked improvements in angina relief and quality of life scores. These benefits were confined to patients with technically successful recanalization and emphasized the clinical relevance of procedural planning, operator skill, and hybrid algorithm implementation.

In regard to procedural safety, this has improved markedly in contemporary practice. Across large registries, perforation rates generally ranged from 1–3%, with slightly higher rates in ADR and retrograde dissection/re-entry cohorts. Mortality and tamponade rates remained below 0.5% even in complex lesions [11,12,20,26]. The integration of imaging, dual access, and controlled dissection tools has contributed substantially to this safety profile. Studies such as Suzuki et al. [12] and Christopoulos et al. [19] confirm that high procedural success can be achieved without increased risk when performed by trained operators within dedicated CTO programs.

Procedure time, fluoroscopy time, and radiation exposure differed significantly among CTO crossing strategies. In the ERCTO registry, AWE (classified as AW-0) had the shortest median procedure time at 66.0 min (IQR: 50.0–90.0) and the lowest radiation exposure with a median dose area product (DAP) of 3.8 Gy·cm^2^ (IQR: 2.4–6.1). ADR cases (AW-2) had longer median procedure time (94.0 min, IQR: 70.0–130.0), higher fluoroscopy time (median 40.0 min, IQR: 27.0–60.0), and increased radiation exposure (median DAP 5.2 Gy·cm^2^, IQR: 3.2–8.2) [18]. These findings were consistent with the PROGRESS-CTO registry, where ADR was associated with significantly longer mean procedure and fluoroscopy times compared to parallel wiring (104 ± 52 vs. 91 ± 46 min; 35 ± 24 vs. 30 ± 20 min, respectively), and higher radiation exposure (4.9 ± 3.2 vs. 4.1 ± 2.8 Gy·cm^2^) [27]. In the OPEN-CTO registry, retrograde procedures exhibited the longest times overall, with a mean procedure duration of 127.6 ± 49.9 min, a mean fluoroscopy time of 50.8 ± 25.4 min, and the highest mean DAP of 6.9 ± 3.9 Gy·cm^2^ [13].

In addition to individual study findings, our meta-analysis synthesizing over 80,000 procedures from 16 studies reinforces the procedural sturdiness of contemporary CTO PCI. The pooled technical success rate of 83.4% and procedural success of 84.6% confirm consistent outcomes across diverse clinical settings and reinforce the reproducibility of hybrid strategies. These figures are remarkably congruent with success rates reported in national and multicenter registries such as BCIS, ERCTO, and PROGRESS-CTO, further supporting their external validity. Moreover, the pooled in-hospital MACE rate of 3.3% reflects an acceptable safety profile despite the technical demands of these interventions. Significantly, MACE incidence remained low even in high-complexity cases employing ADR or retrograde dissection/re-entry techniques, highlighting the impact of operator training, procedural planning, and adjunctive imaging on risk mitigation. All of these meta-synthesis results provide a quantitative foundation for endorsing hybrid CTO PCI as a safe and effective revascularization strategy when appropriately implemented. Similarly, Aljabbary et al. [34] conducted a comprehensive systematic review of 55 observational studies encompassing 28,907 CTO PCI procedures. Their findings support the superior performance of algorithmic strategies—most notably the hybrid approach—with a mean procedural success rate of 87.5% and technical success rate of 88.77%, alongside a low in-hospital mortality of 0.6% and MACE rate of 3.3%. These outcomes compare favorably with single-strategy techniques such as antegrade wire escalation or retrograde-only approaches, and further reinforce the importance of structured, anatomy-driven procedural planning. Moreover, Aljabbary’s review highlights the need for terminology standardization and consistent outcome definitions, echoing calls for greater reproducibility and transparency in CTO PCI literature.

The accumulated evidence affirms CTO PCI as a safe and effective revascularization strategy when guided by hybrid principles, intravascular imaging, and operator expertise. Success rates nearing 90%, coupled with low periprocedural risk and sustained symptomatic benefit, justify CTO PCI as a core component of complete coronary revascularization in appropriately selected patients. The consistent association between successful recanalization and reduced long-term mortality in large registries further supports its clinical relevance.

While this review highlights strong procedural outcomes for hybrid CTO PCI, it is important to contextualize these findings within current international guidelines. Both the 2021 ACC/AHA/SCAI [8] and the 2018 ESC guidelines [35] assign CTO PCI a Class IIb recommendation, reflecting ongoing debate about its prognostic benefit. Large randomized trials such as DECISION-CTO and EuroCTO have demonstrated symptomatic improvement but no significant mortality reduction. This has led to varied adoption in clinical practice, with decisions often influenced by operator expertise, lesion complexity, and institutional policy.

### 4.1. Review Limitations

While this review adhered to PRISMA guidelines and employed rigorous methodology, certain limitations of the review process must be acknowledged. First, the search was restricted to English-language publications and excluded grey literature, introducing a potential for language and publication bias. Second, variability in procedural terminology and outcome definitions across studies posed challenges for data harmonization. Although data extraction and quality assessment were independently performed by multiple reviewers, subjective interpretation could not be entirely eliminated. Moreover, no formal evaluation of publication bias or certainty of evidence using GRADE was conducted, which may limit the generalizability and strength of the synthesized conclusions.

### 4.2. Implications for Clinical Practice and Future Research

The findings of this review have important implications for clinical practice and health policy. The consistently high technical and procedural success rates of hybrid CTO PCI, alongside acceptable safety profiles, support its role as a standard approach for complex coronary revascularization in appropriately selected patients. As operator training, access to adjunctive tools, and procedural standardization improve, broader adoption of hybrid strategies may be warranted. These results may inform updates to interventional cardiology guidelines, particularly regarding procedural algorithms and operator volume thresholds. Future research should prioritize prospective, multicenter randomized trials comparing hybrid CTO PCI to medical therapy in ischemia-driven populations, as well as long-term comparative effectiveness studies of novel wire- and imaging-based re-entry technologies.

## 5. Conclusions

Contemporary CTO PCI, when guided by the hybrid algorithm and supported by adjunctive imaging and re-entry technologies, represents a safe, effective, and reproducible approach across a range of clinical environments. Strategic integration of antegrade and retrograde techniques—tailored to lesion anatomy and procedural response—enhances crossing success while maintaining an acceptable safety profile. Antegrade wire escalation remains the preferred initial strategy, with dissection/re-entry and retrograde approaches serving essential roles in complex or ambiguous occlusions. Beyond technical outcomes, successful CTO recanalization is consistently associated with improved symptom burden, quality of life, and, according to large registries, long-term survival. These results support the role of hybrid CTO PCI as a promising and increasingly adopted revascularization approach in appropriately selected patients, particularly within experienced centers.

## Figures and Tables

**Table 1:** Summary characteristics of included studies.

Study	Country/Registry	Design	*n*	Primary Techniques	Key Devices	Technical/Procedural Success (%)	In-Hospital MACE (%)
Simsek et al. [27]	Multinational	Registry	1725	ADR vs. PW	Stingray, CrossBoss	78/75 (ADR/PW); 75.6/72.6 (Proc)	3.7/1.9
Rempakos et al. [22]	USA & Intl (PROGRESS-CTO)	Registry	12,568	ADR (primary–tertiary)	Stingray, CrossBoss, STAR	77/75.5	3.7 (ADR); 1.6 (non-ADR)
Vadalà et al. [18]	Europe (ERCTO)	Prospective	2395	AWE, ADR, RWE, RDR	Standard CTO sets	93.1 (AWE); 84.5 (ADR); 87.6 (RWE); 82.6 (RDR)	3.4 (ADR); 6.6 (RDR)
Tajti et al. [20]	USA & Intl (PROGRESS-CTO)	Registry	1313	Hybrid (AWE, ADR, RWE, RDR)	NR	90.7/85.2	2.7
Maeremans et al. [11]	Europe (RECHARGE)	Prospective	1177	Hybrid (AWE, ADR, RWE, RDR)	CrossBoss (38%), Stingray (34%)	86/NR	3.6
Christopoulos et al. [19]	USA (PROGRESS-CTO)	Registry	1036	Hybrid (AWE, ADR, Retrograde)	CrossBoss, Stingray, etc.	91/90	1.7
Karmpaliotis et al. [26]	USA (11 centres)	Registry	1301	Antegrade/Retrograde	NR	94/85 (Tech); 93/82 (Proc)	1.1/4.3
Konstantinidis et al. [17]	Europe (ERCTO)	Prospective	17,626	Antegrade, Retrograde, ADR	NR	79.7 → 89.3 (2008–15)	≈5
Sapontis et al. [13]	USA (OPEN-CTO)	Prospective	1000 pts/1054 lesions	Hybrid	CrossBoss, Stingray	86 (core), 90 (op.)/81–85	7.0
Tsai et al. [15]	USA (VA CART)	National registry	2394 pts/2516 lesions	Standard CTO PCI	DES 81%, BMS 19%	79.8/79.7	4.3
Lee et al. [10]	Asia (DECISION-CTO)	RCT	834	Antegrade ± Retrograde (25%)	DES	90.6/≈90	0.9
Suzuki et al. [12]	Japan (41 centres)	Prospective	2846	PAA, PRA, RRA	IVUS, microcatheters	91/87/78 (Tech); 88.8 (Proc)	1.7
Karacsonyi et al. [25]	USA (1 centre)	RCT	246	CrossBoss ADR vs. AWE	CrossBoss, Stingray	87.8/87.1 (Tech); 85.3/83.1 (Proc)	3.3/4.0
Danek et al. [21]	USA (11 centres)	Registry	1313	ADR vs. AWE/Retrograde	CrossBoss, Stingray > 50%	86.9/91.8 (Tech); 85.0/90.7 (Proc)	2.9/2.2
Kinnaird et al. [14]	UK (BCIS)	National	28,050	CTO PCI with enabling strategies	IVUS, atherectomy, etc.	56.8 → 83.8	0.7 → 2.0
Vemmou et al. [28]	Multinational	Pooled analysis	11,961	ISR vs. De novo CTO PCI	IVUS/OCT frequent in ISR	84.9/85.2 (Tech); 83.7/83.9 (Proc)	1.9/2.5
Quadros et al. [29]	Latin America (7 countries)	Registry	1040	AWE 81%, ADR 8%, Retro 11%	Guidewires, IVUS variable	82.5 (range 65–100)	3.1
Mohebbi et al. [24]	Iran (RAIAN)	Single-centre	790	Hybrid (antegrade, ADR, retrograde)	IVUS, microcatheters	70.3/70.3	3.5
Hannan et al. [16]	USA (NY State)	Population registry	4030	CTO PCI (techniques NR)	NR	63.6 (success)	≈3 (MACE)
Kwon et al. [23]	Korea (Asan)	Single-centre	1635	Antegrade/Retrograde	Stenting routine	79.5 → 87.1 (post-retrograde)	4.3/4.1

Note. ADR: antegrade dissection and re-entry; AWE: antegrade wire escalation; BMS: bare-metal stent; CTO: chronic total occlusion; DES: drug-eluting stent; IVUS: intravascular ultrasound; MACE: major adverse cardiac events; OCT: optical coherence tomography; PCI: percutaneous coronary intervention; PRA: primary retrograde approach; PAA: primary antegrade approach; RDR: retrograde dissection and re-entry; RWE: retrograde wire escalation; STAR: subintimal tracking and reentry; VA: Veterans Affairs. Technical success refers to a successful CTO crossing with restoration of TIMI flow and acceptable residual stenosis. Procedural success includes technical success without in-hospital MACE. MACE definitions vary slightly by study but generally include death, MI, stroke, or repeat revascularization.

**Table 2:** Summary of Randomized Controlled Trials Evaluating CTO PCI Strategies.

Trial	Study Question	Intervention(s)	Comparator/Control	Principal Findings and Interpretation
DECISION-CTO [10]	Does CTO PCI improve outcomes compared with optimal medical therapy?	CTO PCI using operator-selected antegrade or retrograde techniques (≈25% retrograde use)	Optimal medical therapy alone	PCI achieved excellent technical success (~90%) with very low periprocedural complication rates. While hard outcomes (death, MI, stroke, revascularization) were similar to medical therapy, PCI provided sustained improvements in angina and quality of life at 1–3 years. High crossover limited statistical power.
CrossBoss First Trial [25]	Does an up-front device-based ADR approach improve efficiency versus standard wire escalation?	CrossBoss/Stingray ADR system	Conventional antegrade wire escalation	Both techniques achieved nearly identical technical and procedural success with low complication rates. No procedural-efficiency advantage was observed, confirming ADR safety but not superiority. The trial validated ADR feasibility in experienced centers.

**Table 3:** Summary of Observational and Registry Evidence on CTO PCI Techniques (2015–2024).

Domain/Representative Studies	Scope and Setting	Dominant Techniques	Key Interpretive Outcomes
Hybrid Strategy Registries [11,13,19,20]	Large multicenter registries across the US and Europe implementing the hybrid algorithm.	Dynamic integration of AWE, ADR, and retrograde methods with real-time switching.	Demonstrated reproducibly high success (~85–90%) and low complication rates (<4%) across diverse centers. Confirmed the hybrid algorithm as the benchmark contemporary framework.
Antegrade Dissection/Re-entry (ADR) [21,22,27]	Multicenter registries assessing ADR role and temporal trends.	CrossBoss/Stingray and STAR techniques used as primary, secondary, or bailout strategies.	ADR yields moderately lower success and slightly higher perforation/MACE than wire escalation but remains indispensable for long, complex, or ambiguous-cap lesions. Use of ADR has declined with improved retrograde proficiency.
Retrograde Approaches [2,12,23]	High-volume centers in the US, Japan, and Korea.	Septal and epicardial collateral tracking, reverse CART, IVUS-guided crossing.	Retrograde adoption increased technical success in complex CTOs without significantly raising periprocedural mortality. Long-term follow-up shows higher re-occlusion risk, underscoring the need for meticulous stent optimization.
Temporal and Geographic Trends [17,24,29]	Continental registries from Europe, Latin America, and the Middle East.	Predominantly hybrid approaches; ADR and retrograde adapted variably by region.	Success rates of 80–90% achieved globally, confirming transferability of hybrid methodology. Resource-limited settings reported slightly lower success but comparable safety.
In-stent CTO PCI [28]	Pooled analysis of 4 major registries.	AWE and imaging-guided wiring predominant.	Immediate outcomes similar to de novo CTOs; however, higher long-term revascularization rates indicate inferior durability of ISR CTO PCI.
Enabling Technologies and Imaging [12,14]	National and expert-center registries.	Dual access, IVUS, atherectomy, microcatheters, CrossBoss/Stingray.	Increasing use of ≥3 enabling tools markedly improved procedural success but modestly raised perforation rates, highlighting the balance between efficacy and complexity.
Population-Based Evidence [15,16]	National datasets from the US VA system and New York State.	Standard real-world CTO PCI, operator-discretional technique.	Demonstrated mortality benefit of successful CTO PCI versus failed attempts and confirmed influence of operator and institutional volume on outcomes.

**Table 4:** Procedural Outcomes Across Included Studies.

Study Type/Setting	Technical Success (%)	Procedural Success (%)	In-Hospital MACE (%)	Perforation (%)	Tamponade (%)	In-Hospital Mortality (%)
Randomized trials (*n* = 2) [10,25]	87–91	83–86	2–4	1–7	≤0.5	≤0.3
Multicenter hybrid registries (*n* = 6) [11,13,17,19,20,21]	85–91	83–90	1.7–3.6	1–3	≤1	≤0.3
ADR-focused analyses (*n* = 2) [22,27]	75–78 (ADR) vs. 88–89 (non-ADR)	73–76 (ADR) vs. 87–88 (non-ADR)	3.7 vs. 1.6–1.9	3–9 (ADR) vs. 4 (non-ADR)	≤0.5	≤0.7
Retrograde strategy studies (*n* = 3) [12,23,26]	78–87	80–85	2–4	2–5	≤1	≤0.3
National/large-scale registries (*n* = 4) [14,15,16,17]	56–89	67–89	2–5	1–4	≤1	0.1–0.9
Developing-region cohorts (*n* = 2) [24,29]	70–83	70–82	3–3.5	≤1	0.4–0.9	0.3–1.0
Overall weighted mean	≈86	≈84	≈2.8	≈2.6	≈0.6	≈0.4

**Table 5:** Procedural Outcomes by Crossing Strategy.

Strategy	Mean Technical Success (%)	Mean Procedural Success (%)	Typical MACE (%)	Typical Perforation (%)	Distinguishing Features
AWE	90–93	85–90	1.5–2.5	1–2	High success in less complex lesions; baseline strategy in most cases.
ADR	75–86	72–85	3–5	3–9	Used as secondary strategy; greater radiation, contrast, and perforation risk.
RWE	85–88	80–85	2–4	2–4	Effective for proximal cap ambiguity or long lesions; requires dual access.
RDR	78–83	75–80	3–6	5–7	Highest fluoroscopy and contrast use; reserved for complex and refractory occlusions.
Hybrid (Integrated AWE + ADR + Retrograde)	86–91	83–89	1.5–3.5	2–3	Combines approaches within one procedure; consistent success across registries.
Enabling Techniques (IVUS, atherectomy, dual access)	+10–15 improvement in success vs. baseline	–	↓ complications when used systematically	–	Improves planning and wire control; associated with 83–84% national success in BCIS data [14]

## Data Availability

No new data were created or analyzed in this study. Data sharing is not applicable to this article.

## Supplementary Materials

The following supporting information can be downloaded at: https://www.mdpi.com/article/10.3390/life15111739/s1, Supplementary Table S1: Full search strategies for PubMed, Cochrane CENTRAL, and Google Scholar.

## Abbreviations

The following abbreviations are used in this manuscript:

ADR	Antegrade Dissection and Reentry
AFR	Antegrade Fenestration and Reentry
AWE	Antegrade Wire Escalation
BASE	Balloon-Assisted Subintimal Entry
CABG	Coronary Artery Bypass Grafting
CAD	Coronary Artery Disease
CART	Controlled Antegrade and Retrograde Tracking
CTO	Chronic Total Occlusion
HDR	Helical Dissection Re-entry
ISR	In-Stent Restenosis
IVUS	Intravascular Ultrasound
MACE	Major Adverse Cardiac Events
PAA	Primary Antegrade Approach
PCI	Percutaneous Coronary Intervention
PRA	Primary Retrograde Approach
RDR	Retrograde Dissection and Re-entry
Reverse CART	Reverse Controlled Antegrade and Retrograde Tracking
RFR	Reentry with Focal Reentry
RWE	Retrograde Wire Escalation
Side-BASE	Side-Branch Assisted Subintimal Entry
STAR	Subintimal Tracking and Reentry
STEMI	ST-Elevation Myocardial Infarction
TIMI	Thrombolysis in Myocardial Infarction
TVF	Target Vessel Failure
TVR	Target Vessel Revascularization
