# Supplementary material for: Hybrid Strategies for CTO PCI: A Systematic Review and Meta-Analysis of Antegrade and Retrograde Techniques
Source: Life (Basel). 2025 Nov 12;15(11):1739. doi: 10.3390/life15111739 (PMC12654039; doi:10.3390/life15111739)
Supplement: Supplementary file 1 [file life-15-01739-s001.zip › life-3951983-supplementary.pdf]

Supplementary Table S1 – Full Search Strategy

This supplementary table provides the complete search strategies used for each database in the systematic review titled " Hybrid Strategies for CTO PCI: A Systematic Review and Synthesis of Antegrade and Retrograde Techniques." Searches were conducted from January 2015 to June 2025.

| Database | Search Strategy |
|---|---|
| PubMed | ("chronic total occlusion"[Title/Abstract] OR "CTO"[Title/Abstract]) AND ("percutaneous coronary intervention"[Title/Abstract] OR "PCI"[Title/Abstract]) AND ("antegrade dissection"[Title/Abstract] OR "ADR"[Title/Abstract] OR "AFR"[Title/Abstract] OR "RFR"[Title/Abstract] OR "BASE"[Title/Abstract] OR "Side-BASE"[Title/Abstract] OR "HDR"[Title/Abstract] OR "antegrade wire escalation"[Title/Abstract] OR "retrograde"[Title/Abstract] OR "CART"[Title/Abstract] OR "reverse CART"[Title/Abstract] OR "retrograde wire escalation"[Title/Abstract] OR "confluent ballooning"[Title/Abstract]) AND ("technical success"[Title/Abstract] OR "procedural success"[Title/Abstract] OR "complication"[Title/Abstract] OR "adverse event"[Title/Abstract] OR "MACE"[Title/Abstract] OR "mortality"[Title/Abstract]) |
| Cochrane CENTRAL | ("chronic total occlusion" OR CTO) AND ("percutaneous coronary intervention" OR PCI) AND ("antegrade dissection" OR ADR OR AFR OR RFR OR BASE OR Side-BASE OR HDR OR "antegrade wire escalation" OR retrograde OR CART OR "reverse CART" OR "retrograde wire escalation" OR "confluent ballooning") AND (success OR outcome OR complication OR MACE OR mortality) |
| Google Scholar | "chronic total occlusion" AND PCI AND (antegrade dissection OR ADR OR AFR OR RFR OR BASE OR Side-BASE OR HDR OR "antegrade wire escalation" OR retrograde OR CART OR "reverse CART" OR "retrograde wire escalation" OR "confluent ballooning") AND (success OR complication OR outcome OR MACE OR mortality) |
| Manual search | Manual hand-searching of bibliographies, reference lists of included studies and guidelines. |
